# Chitosan-Dextran-Glycerol Hydrogels Loaded with Iron Oxide Nanoparticles for Wound Dressing Applications

**DOI:** 10.3390/pharmaceutics14122620

**Published:** 2022-11-28

**Authors:** Cristina Chircov, Iuliana Teodora Bejenaru, Adrian Ionuț Nicoară, Alexandra Cătălina Bîrcă, Ovidiu Cristian Oprea, Bianca Tihăuan

**Affiliations:** 1 Department of Science and Engineering of Oxide Materials and Nanomaterials, University Politehnica of Bucharest, 011061 Bucharest, Romania; 2 National Research Center for Micro and Nanomaterials, University Politehnica of Bucharest, 060042 Bucharest, Romania; 3 Faculty of Medical Engineering, University Politehnica of Bucharest, 060042 Bucharest, Romania; 4 Department of Inorganic Chemistry, Physical Chemistry and Electrochemistry, University Politehnica of Bucharest, 1-7 Polizu St., 011061 Bucharest, Romania; 5 Research Institute of the University of Bucharest—ICUB, 91-95 Splaiul Independentei, 50567 Bucharest, Romania; 6 Research & Development for Advanced Biotechnologies and Medical Devices, SC Sanimed International Impex SRL, 087040 Călugăreni, Romania

**Keywords:** chitosan, dextran, glycerol, iron oxide nanoparticles, hydrogels, wound dressings

## Abstract

Natural polymers have shown tremendous potential towards the development of hydrogels with tissue regeneration properties. Among them, chitosan and dextran are polysaccharides widely applied in the wound dressing area owing to their mucoadhesiveness, biodegradability, hemostatic potential, and intrinsic antibacterial activity, while glycerol is a well-known biocompatible solvent extensively used in the manufacture of cosmetic, pharmaceutical, medical, and personal care products. In order to enhance the properties of natural polymer-based hydrogels, the focus has currently shifted towards the addition of nanomaterials with antibacterial and regenerative potential, i.e., iron oxide nanoparticles. Thus, the aim of the present study was to develop a series of chitosan-dextran-glycerol hydrogels loaded with iron oxide nanoparticles, either readily added or formed in situ. The physicochemical properties of the so obtained hydrogels demonstrated an improved dispersibility of the in situ formed magnetite nanoparticles, which further decreases the porosity and swelling ratio of the hydrogels but increases the antimicrobial properties. Additionally, the presence of glycerol enhances the cell viability but reduces the antimicrobial potential. In this context, the results proved promising biological and antimicrobial properties, thus confirming their potential as biomaterials for wound healing and regeneration.

## 1. Introduction

With a surface area of ~1.8 m^2^, the skin represents the largest organ of the human body and, although it is only 1.5–4 mm thick, it accounts for 10–15% of the total body weight [1,2]. Since it plays a series of essential roles for the well-being of the human body, including acting as a physical and immunological active barrier, body temperature regulator, sensory receptor, water balance, and vitamin and hormone synthesis [3,4], maintenance of its structural and functional integrity is fundamental. However, the prevalence of burn injuries is still a major cause of concern, with 11 million cases reported annually worldwide, of which 180,000 are fatal [5]. Furthermore, since the elderly population is constantly growing, it is expected that chronic wounds will concomitantly be an increasingly persistent problem [6]. Therefore, in order to increase the life quality of patients suffering from acute or chronic wounds and to consequently limit the economic burden posed upon the health care system [7], it is necessary to develop novel methods that enhance wound healing and skin regeneration processes.

The current strategy in wound management focuses on the development of hydrogels consisting of biomaterials with regenerative properties. Hydrogels represent three-dimensional cross-linked polymeric networks, capable of absorbing large amounts of water while maintaining their hierarchical structures [8,9,10,11,12]. Within the wound healing direction, hydrogels are able to incorporate and release a wide variety of bioactive compounds and absorb excess fluid while maintaining a moist microenvironment [13]. Generally, natural polymers are preferred for the fabrication of hydrogels owing to their increased biocompatibility and biodegradability, e.g., chitosan, dextran, gelatin, cellulose, alginate, hyaluronic acid [14,15]. Furthermore, besides ensuring the promotion of the wound healing process, hydrogels can also prevent the occurrence of infections at the wound site. Therefore, besides the intrinsic antimicrobial properties of the constituent polymers, the anti-infective potential of the hydrogels could be enhanced through the addition of nanoparticles to ensure a synergistic effect.

In this context, the present study aimed to develop a series of composite hydrogels for wound dressing applications, that could ensure both the regeneration of the skin and the prevention of infection occurrence. Specifically, the continuous phase of the hydrogels consisted of chitosan, dextran, and glycerol, while the dispersed phase included either readily added or in situ formed magnetite nanoparticles. On one hand, chitosan is one of the most abundant biopolymers found in nature, and is a linear heteropolysaccharide composed of various amounts of D-glucosamine and N-acetyl-D-glucosamine units linked through (β1→4) glycosidic bonds [16,17,18]. It has been widely investigated in the biomedical sector owing to its antioxidant, antimicrobial, and regenerative properties [19]. On the other hand, dextran is a complex branched polysaccharide composed of α-1,6-linked d-glucopyranose of microbial origins [20,21]. The biomedical applications of dextran focus on tissue engineering scaffolds owing to its anti-thrombolytic and bio-adhesive properties [22]. Moreover, glycerol is a biocompatible and water-soluble polyol compound commonly used in the cosmetic and pharmaceutical industries for moisture control [23].

Thus, the components of the obtained hydrogels would promote wound healing and regeneration processes, while also preventing the occurrence of infections through the synergistic action of chitosan and magnetite.

## 2. Materials and Methods

### 2.1. Materials

The materials used within the present study included ferric chloride hexahydrate (FeCl_3_·6H_2_O), ferrous sulfate heptahydrate (FeSO_4_·7H_2_O), sodium hydroxide (NaOH), chitosan (medium molecular weight, M_w_ = 190–310 kDa), dextran (M_w_ = 500 g/mol), D-raffinose pentahydrate, acetic acid, which were purchased from Sigma-Aldrich Merck (Darmstadt, Germany), and glycerol, from Carlo Erba Reagents (Val-de-Reuil, France). All chemicals were of analytical purity and used with no further purification.

### 2.2. Synthesis Methods

Magnetite nanoparticles were obtained through the co-precipitation method. Briefly, the solution containing FeCl_3_·6H_2_O and FeSO_4_·7H_2_O (2:1 molar ratio) was dripped into a 1 M NaOH solution using a peristaltic pump. Using a high-power permanent magnet, the nanoparticles were decanted and rinsed with deionized water until a neutral pH.

For the synthesis of the composite hydrogels, a stock solution was obtained by dissolving chitosan at the concentration of 6% in a 2% acetic acid solution, followed by the addition of 1% D-raffinose pentahydrate as a thickening agent. Subsequently, two solutions of dextran 30% were obtained by dissolving in deionized water and glycerol, followed by the addition of magnetite nanoparticles or magnetite precursors at three different concentrations (1, 5, and 10%). Finally, 10 mL of the chitosan solution and 1 mL of the dextran solution were mixed, poured into Petri dishes, and pulverized with NaOH 8% until solidification. The so obtained hydrogels were rinsed with deionized water until they reached a neutral pH and lyophilized (Figures S1 and S2, Table 1).

### 2.3. Characterization Methods

#### 2.3.1. X-ray Diffraction (XRD)

A PANalytical Empyrean diffractometer (PANalytical, Almelo, The Netherlands) equipped with a CuKα radiation was used for the XRD analysis. The hydrogels were scanned within the 10–80° 2θ angle range, at an incidence angle of 0.5°, a step size of 0.0256°, and the time per step of 1 s.

#### 2.3.2. Fourier Transform Infrared Spectroscopy (FT-IR)

The IR spectra were acquired using a Thermo Scientific Nicolet iS50 (Thermo Fischer Scientific, Waltham, MA, USA) spectrometer equipped with a liquid nitrogen-cooled mercury cadmium telluride detector. Measurements were performed using the attenuated total reflectance (ATR) mode, in the range of 4000–400 cm^−1^ and at a resolution of 4 cm^−1^, at room temperature. For each sample, the OmnicPicta (8.2.0.387 Thermo Nicolet, Thermo Fischer Scientific, Waltham, MA, USA) was used for the co-addition and processing of 64 scans.

#### 2.3.3. Scanning Electron Microscopy (SEM) and Energy Dispersive X-ray Spectroscopy (EDX)

The morpho-structural characteristics of each hydrogel were analyzed in cross-section through SEM, by fixating a small piece onto a carbon band that was placed inside the analysis chamber of the Inspect F50 high-resolution microscope, coupled with an energy dispersive spectrometer (Thermo Fisher—former FEI, Eindhoven, The Netherlands). SEM images were acquired using secondary and backscattered electrons with 30 KeV energy at different magnifications. In order to ensure the quality of the micrographs, all samples were coated with a nanosized layer of gold.

#### 2.3.4. Thermogravimetry and Differential Scanning Calorimetry (TG-DSC)

An STA TG/DSC Netzsch Netzsch Jupiter 449 F3 equipment (Selb, Germany) was used for the TG-DSC analysis. The temperature ranged between 20 and 900 °C in a dynamic atmosphere of 50 mL/min air with a heating rate of 10 K/min in an alumina crucible.

#### 2.3.5. Porosity and Swelling Degree

The porosity of the hydrogels was determined in 1 mL of water for each sample (V_0_) by measuring the volume when the hydrogel was immersed (V_1_) and after its removal (V_2_). The porosity was calculated using the following equation:

(1) $$\mathrm{Porosity}\;(\%)=\frac{V_{0}-V_{2}}{V_{1}-V_{2}}\times 100$$

The swelling ratio of the hydrogels was determined in simulated body fluid (SBF) from 1 min to 21 days. The lyophilized hydrogels were weighed in order to establish the initial mass (M_0_), followed by repeated immersion, removal, and weighing at predetermined time points (M*_t_*). The swelling degree was calculated using the following equation:

(2) $$\mathrm{Swelling}\;\mathrm{degree}\;(\%)=\frac{M_{t}-M_{0}}{M_{0}}\;\times \;100$$

#### 2.3.6. Cell Viability

The MTT assay was performed on a HUVEC—human umbilical vein endothelial cell line. Prior to the testing, all samples were sterilized for 1 h using UV light. The samples were tested in the form of 6 mm round pieces at two time points, i.e., 24 and 48 h. After removing the culture medium, the cells were washed with PBS and dark-incubated for 2 h with (3-(4,5-dimethylthiazol-2-yl)-2,5-diphenyltetrazolium bromide solution at 37 °C and 5% CO_2_. After removing the MTT solution, the formazan crystals were resuspended in isopropanol until complete solubilization. The absorbance was spectrophotometrically determined at 595 nm using a SpectraMax i3x Multi-Mode microplate reader and the SoftMax Pro 6 software. The cell viability was calculated using the following equation:

(3) $$\mathrm{Cell}\;\mathrm{viability}\;\left(\%\right)=\frac{\mathrm{OD}595\;\mathrm{sample}}{\mathrm{OD}595\;\mathrm{control}}\;\times \;100$$

#### 2.3.7. Antimicrobial Activity

Assessment of antimicrobial activity was performed using three reference microorganisms by American Type Culture Collection (ATCC, Manassas, VA, USA): *Staphylococcus* (S.) *aureus* ATCC 6538, *Pseudomonas* (Ps.) *aeruginosa* ATCC 9027, and *Candida* (C.) *albicans* ATCC 10231. Microbial susceptibility was assessed according to CLSI 2019 M100 [24]. Samples (Table 1) were cropped into 6 mm disks and incubated at 36 ± 2 °C for 4 h with 10^5^ microbial suspensions obtained from 1.5 × 10^8^ CFU/mL bacterial suspensions (standard density of 0.5 McFarland) and 3.0 × 10^8^ CFU/mL microfungi suspensions (1 McFarland). After 4 h of incubation, the samples were thoroughly spun on a vortex (1000 rpm) for 30 min, and 6 decimal serial dilutions were carried out. 10 µL from each dilution were plated on solid Mueller Hinton agar (Liofilchem, Italy) and Sabouraud (Liofilchem, Italy) for microfungi. After 24 h of incubation, the colonies were counted. Antimicrobial activity (AA) was expressed as a logarithmic reduction using the formula:

(4) $$\mathrm{Antimicrobial}\;\mathrm{activity}\;(\mathrm{CFU}/\mathrm{mL})=\frac{\mathrm{lgA}}{\mathrm{lgB}}$$

where A is CFU/mL of negative control (initial number of untreated bacteria in the inoculum); B is CFU/mL of samples (number of bacteria after x time of contact with antimicrobial substance). The logarithmic reduction (L) was converted to percentage reduction using the formula:

(5) P (%)=(1 - 10 - L) × 100

#### 2.3.8. Statistical Analysis

The statistical analysis was performed using GraphPad Prism 9 (San Diego, CA, USA). Data were analyzed using One Way Anova (*n* = 3; the level of significance set to *p* < 0.05).

## 3. Results

The present study aimed to obtain a series of composite hydrogels that could enhance the skin regeneration process, and ensure an antimicrobial activity that would prevent the occurrence of infections at the wound site. In this context, four types of hydrogels were obtained, where the continuous phase included either chitosan and dextran or chitosan, dextran, and glycerol. The dispersed phase consisted of either previously synthesized magnetite nanoparticles or iron precursors that would lead to the in situ formation of magnetite.

**Figure 1:**
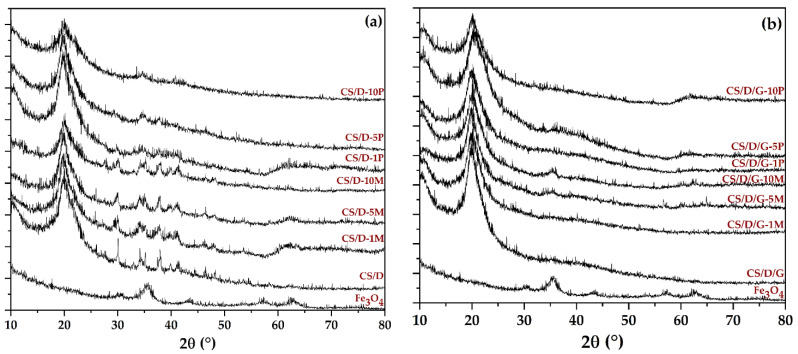
Diffractograms acquired for the CS/D (**a**) and CS/D/G (**b**) hydrogels.

The first analysis that we employed consisted of XRD in order to determine the phases present within the hydrogels (Figure 1). The presence of magnetite within both readily added or in situ formed magnetite is demonstrated through the decrease of the diffraction peaks corresponding to chitosan and dextran, and the increase of the major peak characteristic to magnetite (2θ angle of 35°). The absence of most diffraction peaks within the diffractograms corresponding to the in situ formed magnetite could be associated with the formation of complexes between the iron ions and the polymers present within the hydrogels. Thus, it could be assumed that CS/D-P hydrogels contain mixtures of magnetite nanoparticles and iron atoms bonded to the polymeric chains. Moreover, the presence of glycerol further diminishes the intensity of the diffraction peaks associated with both magnetite and the added polymers. This behavior would be expected, since the lyophilization process did not lead to the removal of glycerol from the hydrogels as it did with the water molecules.

**Figure 2:**
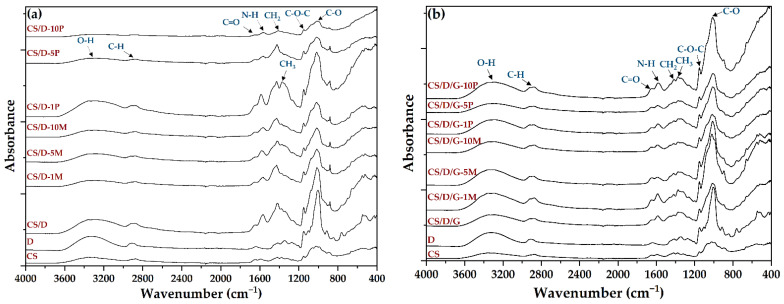
FT-IR spectra of the CS/D (**a**) and CS/D/G (**b**) hydrogels.

Subsequently, the composite hydrogels, as well as the chitosan and dextran powders, were subjected to the FT-IR analysis in order to determine the functional groups present (Figure 2, Table 2). All spectra present absorption peaks specific for O-H stretching and intramolecular hydrogen bonds between 3600 and 3100 cm^−1^ [25]. However, it can be seen that within the CS/D hydrogels, the intensity of the band decreases with the increase in the concentration of added or formed magnetite. In contrast, the addition of glycerol preserves the intensity of the signal due to the high amount of hydroxyl groups present within its molecule. Furthermore, the bands at ~2917 and ~2877 cm^−1^ correspond to the C-H symmetric and C-H asymmetric stretching, respectively, which are generally found in the spectra of various polysaccharides [26,27]. Additionally, both dextran and chitosan exhibit bands at ~1417, ~1370, ~1149, and 1012 cm^−1^ which are characteristic for the CH_2_ bending, CH_3_ symmetrical deformation, asymmetric C-O-C stretching, and C-O stretching, respectively [27]. The spectra also present bands specific for the C=O stretching within amide I at 1651 cm^−1^ and the N-H bending specific for primary amines at 1567 cm^−1^ [27,28]. Although magnetite presents an absorption band at ~541 cm^−1^ [29,30,31,32], the signal corresponding to the Fe-O bond might be covered by the dextran-specific absorption bands.

Hydrogels were further subjected to the SEM analysis in order to investigate the morphology of the pores and the presence of the nanoparticles on the pore surface. For the control CS/D and CS/D/G hydrogels, micrographs were acquired using an ETD detector, while for the hydrogels containing readily added or in situ formed magnetite, a CBS detector was used for differentiating between heavy and light elements. 

**Figure 3:**
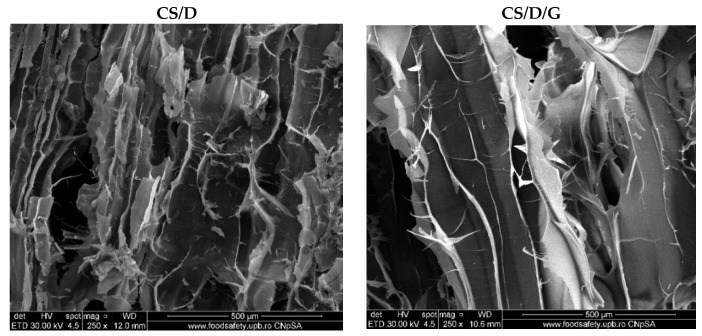
SEM images of the control CS/D and CS/D/G hydrogels (ETD detector).

As can be seen, the control hydrogels (Figure 3) exhibit an interconnected porous structure, which is essential for the processes of cell migration and transport of biomolecules required for skin regeneration. Moreover, the pores of both hydrogels exhibit a lamellar structure with ranging dimensions. Specifically, the pore diameter of the CS/D hydrogels varies between 20 and 150 nm, while for the CS/D/G, the pore size is ~150 nm. Therefore, it can be concluded that the presence of glycerol within the composite hydrogels leads to the formation of larger, but more uniform internal pores.

**Figure 4:**
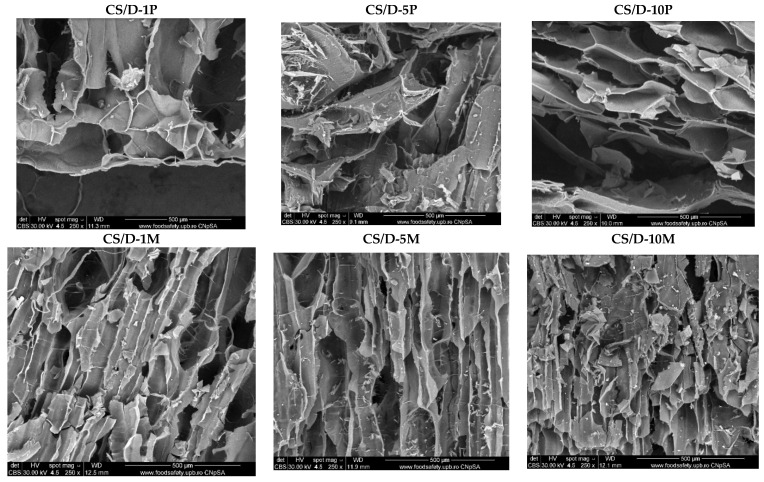
SEM images of the CS/D hydrogels (CBS detector).

Figure 4 depicts the SEM micrographs obtained for the CS/D hydrogels containing readily added or in situ formed magnetite. It can be seen that the addition of magnetite within the hydrogels does not modify the structure of pores, which are similar to the control. Furthermore, the presence of concentration-dependent amounts of agglomerates with varying sizes of 4–25 μm consisting of magnetite nanoparticles can be observed on the entire surface of the pores. In contrast, the addition of iron precursors leads to the modification of the pore shape, which becomes polyhedral. The presence of agglomerates with distributions increasing with the increase of the precursor concentration can be observed. However, there are no noticeable agglomerates within the CS/D-10P hydrogel, probably due to the internalization of the iron ions within the pore walls and the formation of iron complexes with the polymeric chains.

**Figure 5:**
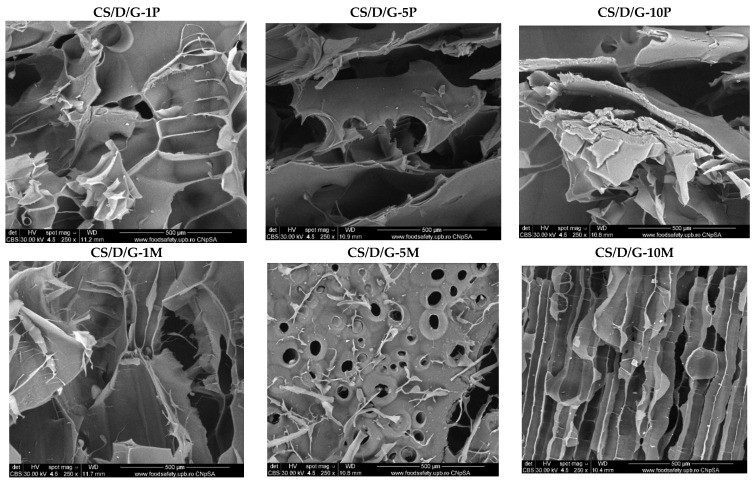
SEM images of the CS/D/G hydrogels (CBS detector).

Subsequently, the micrographs for the CS/D/G hydrogels containing readily added or in situ formed magnetite are presented in Figure 5. Similar to the CS/D hydrogels, the presence of agglomerates increases with the concentration of magnetite nanoparticles or iron precursors added within the hydrogels. Anew, the 10% iron precursor concentration leads to the internalization of the iron ions within the pore walls. However, the presence of glycerol leads to the formation of pores with irregular shapes. Furthermore, the CS/D/G-5P and CS/D/G-10P hydrogels do not exhibit a porous structure.

**Figure 6:**
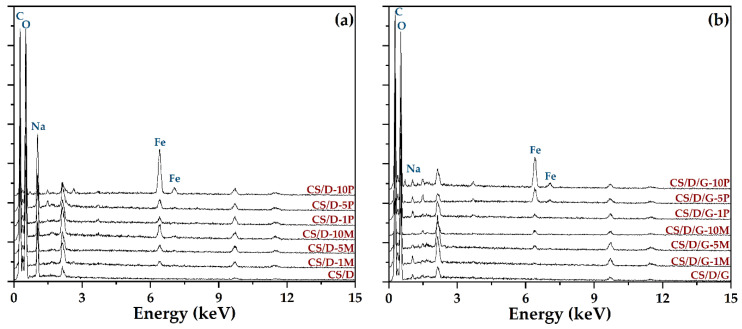
EDX spectra of the CS/D (**a**) and CS/D/G (**b**) hydrogels.

Subsequently, the hydrogels were subjected to the EDX analysis in order to identify the elements present and to determine their concentration (Figure 6). As the spectra show, the intensity of the peak characteristic for iron increases with the concentration of either readily added or in situ formed magnetite. Moreover, the intensity is considerably higher for the hydrogels containing iron precursors, which further confirms their internalization within the hydrogel walls and the possibility to form iron complexes with the polymeric chains. The unmarked peaks are attributed to gold, which was used for coating the hydrogels necessary for the investigation. In this manner, the previously obtained results were confirmed. The distribution of iron within the hydrogels was also determined by elemental mapping (Figures S3 and S4). For the hydrogels containing readily added magnetite nanoparticles, iron can be observed only within the agglomerates found on the surface of the pores. In contrast, within the hydrogels where the iron precursors were introduced in order to generate the in situ formation of magnetite, iron is uniformly distributed throughout the entire hydrogel. Thus, the presence of mixtures of magnetite nanoparticles and iron atoms bonded to the polymeric chains within these types of hydrogels is further confirmed.

**Figure 7:**
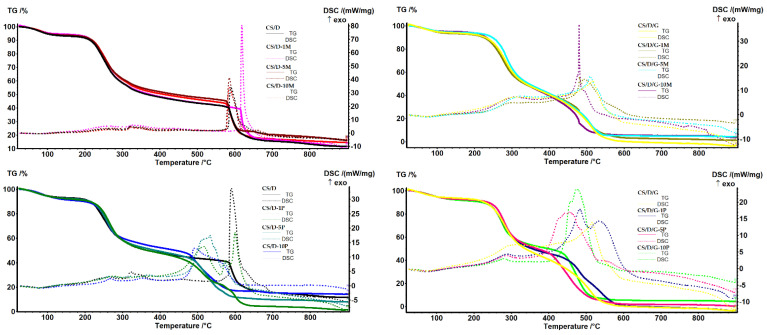
TG-DSC curves for the CS/D and CS/D/G hydrogels.

The TG-DSC analysis was performed in order to determine the mass loss and the associated thermal effects of the hydrogels after thermal treatment (Figure 7). In the case of hydrogels containing readily added magnetite, both CS/D and CS/D/G, the mass loss decreases with the increase of magnetite concentration. Until the temperature of ~150 °C, the water molecules attached to the polymeric chains through hydrogen bonds are removed, leading to an endothermal effect with the minimum at ~80 °C. Further, the organic phase is subjected to oxidative degradation, leading to the fragmentation and subsequent oxidation of the polymeric chains. The process is accompanied by several exothermal effects between 250 and 500 °C for the CS/D hydrogels, and between 300 and 415 °C for the CS/D/G hydrogels. The residual carbonic mass of the CS/D hydrogels is completely oxidized after 575 °C, while the CS/D/G hydrogels are completely oxidized at ~500 °C. In the case of hydrogels containing in situ formed magnetite, the previous mass loss trend is maintained only for the CS/D/G hydrogels. This could be explained by a more homogenous distribution of the in situ formed magnetite nanoparticles owing to the presence of glycerol as the solvent. In regard to the thermal processes that occur, both types of hydrogels behave similarly to the ones containing readily added magnetite.

**Figure 8:**
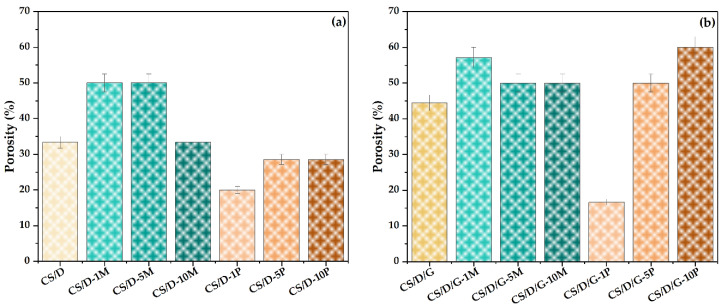
Porosity values for the CS/D (**a**) and CS/D/G hydrogels (**b**).

**Figure 9:**
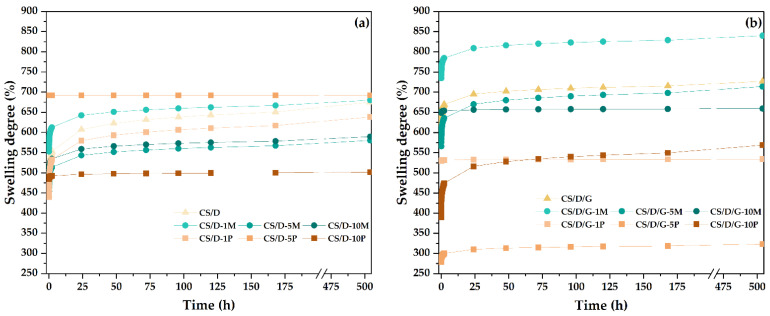
The evolution of swelling degrees of the CS/D (**a**) and CS/D/G hydrogels (**b**).

The final physicochemical characterization involved the investigation of the hydrogel properties in terms of porosity (Figure 8) and swelling degree (Figure 9). First, it can be seen that the use of glycerol as the solvent for dextran leads to an increase in the porosity of the hydrogels. However, since the SEM images show a lack of pores within these types of hydrogels, it can be assumed that the high porosity values are due to the existence of larger void spaces between the polymeric chain network that are not perceived as pores. Second, it can be seen that the addition of magnetite nanoparticles leads to an increase of the hydrogel porosity in comparison to the control hydrogel; however, higher nanoparticle concentrations slightly decrease the porosity of the hydrogels. Third, in the case of hydrogels containing in situ formed magnetite nanoparticles, the porosity differs depending on the presence of glycerol. Specifically, the addition of 1% magnetite precursors decreases the porosity up to two-fold and three-fold for the CS/D and CS/D/G hydrogels, respectively. However, the 5 and 10% concentrations lead to higher porosities, especially for the hydrogels containing glycerol.

The swelling degree was investigated between the time intervals of 1 min and 21 days. In most cases, the swelling degree seems to stagnate after 24 h; nonetheless, the hydrogels containing in situ formed magnetite, i.e., CS/D-5P, CS/D-10P, CS/D/G-1P, CS/D/G-5P, reached the maximum swelling degree in the first minutes after immersion. Moreover, it can be seen that in the case of CS/D hydrogels, the swelling degree for the hydrogels containing 1% readily added magnetite or 1 and 5% in situ formed magnetite are similar to the control, while the concentrations of 5 and 10% or 10%, respectively, decrease the capacity of the hydrogels to absorb fluids. These results are in correlation with the previously determined porosity values. In contrast, in the case of CS/D/G hydrogels, the highest swelling degree values were registered for the 1% readily added magnetite, the 5 and 10% concentrations being similar to the control, which also confirm the porosity values. The swelling degrees of the CS/D/G-P are lower than the control, with the highest value registered for the 1 and 10% concentrations, and the lowest for the 5%. In this manner, the SEM observations regarding the lack of an interconnected porous structure within these hydrogels are confirmed. Moreover, the swelling degrees of the CS/D hydrogels vary between 450 and 700%, while of the CS/D/G hydrogels they vary between 300 and 800%, thus proving the capacity of glycerol to distribute the iron ions more homogenously within the hydrogel network.

**Figure 10:**
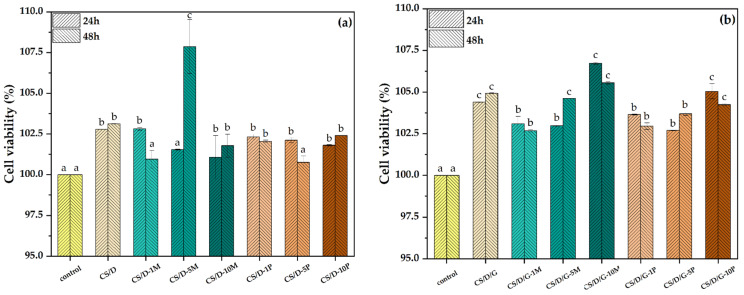
Cell viability for the CS/D (**a**) and CS/D/G (**b**) hydrogels (*p* value < 0.03; different letters indicate levels of significance between each sample).

The cell viability results obtained from the MTT assay performed on the HUVEC cell line is presented in Figure 10. It can be seen that all types of hydrogels lead to an increase of cell viability in comparison to the control, thus proving the biocompatible nature of each hydrogel component. Moreover, the addition of glycerol increases the cell viability of all types of hydrogels, which further demonstrates its well-known biocompatibility. Except for the CS/D-5M hydrogel, there are no significant differences between the two time intervals at 24 and 48 h. Thus, it could be assumed that the 48-h time point is not sufficient for the degradation of the hydrogel and, consequently, the release of magnetite nanoparticles and/or iron ions. Among the two hydrogel types, the highest cell viability values were registered for the CS/D-5M and CS/D/G-10M hydrogels, which demonstrates the regenerative capacity of magnetite nanoparticles. Furthermore, there are no significant differences between the concentrations of magnetite precursors added, which could mean that the release of iron ions is much slower than that of the magnetite nanoparticles.

**Figure 11:**
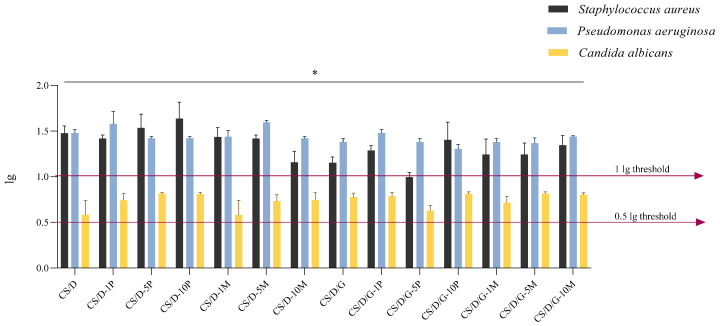
Antimicrobial activity expressed as logarithmic reduction (lg) for hydrogel samples on three reference microorganisms; * *p* value < 0.03 (*n* = 3).

The final investigation employed within the present study involved assessing the antimicrobial activity of the composite hydrogels (Figure 11). Results obtained in this study indicate a high antimicrobial efficacy of hydrogel samples on bacterial strains *S. aureus* and *Ps. aeruginosa.* All samples surpassed the 1 lg threshold marked for the Gram-negative and Gram-positive strains. The antifungal activity of samples was tested on *C. albicans,* and results indicate a moderate activity, with lg reduction surpassing the 0.5 threshold. With the purpose of determining the optimal antimicrobial activity between microorganisms, we marked the best variant of each category in Table 3.

Since *Ps. aeruginosa* is an opportunistic Gram-negative strain directly responsible for the impairment of wound repair processes [33] and causative of local to systemic infections, it is possibly one of the most difficult pathogens among the other tested strains to manage at the wound site (due to its pathogenicity, high prevalence, and rapid colonization of medical devices). The antimicrobial effect obtained by the C/D hydrogels is a promising and favorable therapeutic approach for the management of site infections.

**Figure 12:**
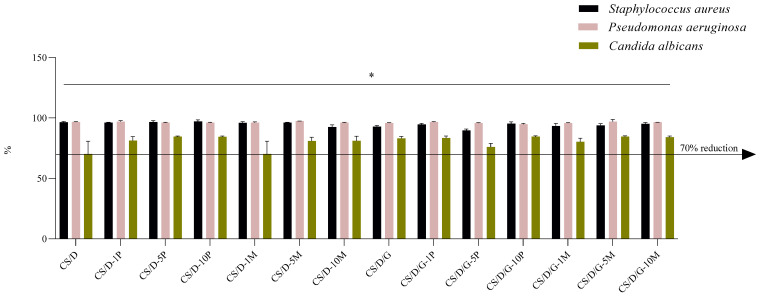
Logarithmic reduction (lg) converted as percentage reductions for hydrogel samples on three reference microorganisms; * *p* value < 0.02 (*n* = 3).

The correspondent percentage reduction resulting from the conversion of lg depicts in greater detail the antimicrobial efficacy of samples (Figure 12). The 70% threshold is analog to the 0.5 lg reduction. For samples CS/D-10P, CS/D-5M, CS/D/G-10P, and CS/D/G-5M, over 90% reduction was obtained on bacterial strains and over 80% reduction on the microfungal strain (Table 4). Therefore, the results obtained indicate the capacity of the samples to interfere with the proliferation and growth of the microbial organisms.

Although in some instances the antimicrobial effect of the hydrogels was not significantly increased by comparison with the control hydrogels (CS/D and CS/D/G), this may be due to the limited exposure time (4 h) to the media, and the slow release of magnetite nanoparticles (an advantageous aspect for long time exposure of hydrogels to the wound site), but also due to the intrinsic high antimicrobial activity of the chitosan-dextran matrix, as reported by others as well [34,35,36,37].

## 4. Discussion

The present study aimed to develop a series of composite hydrogels that could concomitantly enhance the skin regeneration process while ensuring an efficient antimicrobial activity. For this purpose, the continuous phase of the hydrogels included chitosan, a natural polymer with intrinsic antimicrobial and regenerative properties, dextran, a natural polysaccharide with anti-inflammatory capacity, and glycerol, which is a biocompatible solvent widely used for pharmaceutical and cosmetic development. 

Chitosan has proven an antimicrobial activity against a wide spectrum of microbial species, including Gram-positive and Gram-negative bacteria such as *N. gonorrhoeae* [38], *Salmonella* spp. [39], *E. coli* [39,40,41,42], *S. aureus* [40,41,42], *P. aeruginosa* [40,41], *B. subtilis* [41], *B. cereus* [42], *L. monocytogenes* [42], viruses, e.g., norovirus, calcivirus [43], coronavirus [44], fungi such as *C. albicans* [40,45], *C. auris* [40], *A. flavus* [40], *A. albicans* [40], *C. tropicalis* [45], parasites, e.g., *G. lamblia* [46], *Hymenolepis nana* [47], both as hydrogel and nanoparticle formulations. The mechanisms behind the antimicrobial activity of chitosan depends on the microbial species, which are generally associated with differences in the cell wall components [48]. In the context of the antibacterial action of chitosan, the pathway involves the disruption of the cell wall through electrostatic interactions: in Gram-positive bacteria, the positively charged chitosan interacts with the negative charges given by teichoic acid in peptidoglycans, while in Gram-negative bacteria [49,50], it neutralizes the negative charges of lipopolysaccharides and disrupts the outer membrane [51]. In the case of fungal species, chitosan binds to the phosphorylated mannosyl side, which further disrupts the plasma membrane and leads to intracellular material leakage [52,53]. Additionally, other studies demonstrated the pH- and temperature-responsive release of diclofenac from a poly(N-(4-aminophenyl) methacrylamide))-carbon nano-onions/diclofenac-complex integrated chitosan nanocomposite hydrogel nanoparticles system with improved cell viability and swelling degrees between 200% and 450% [54]. Within the scope of the present study, the results demonstrated the intrinsic antimicrobial activity of the continuous phase consisting of chitosan and dextran, which is in accordance with the previously described reports. 

Furthermore, dextran is a biopolymer which has been widely used in the synthesis of composite hydrogels for wound dressings, owing to its capacity to limit inflammation and promote angiogenesis and fast healing [55,56]. While it is generally applied in combination with other polymers such as gelatin [57], cellulose [58,59], chitosan [60,61], hyaluronic acid [55], and polyvinyl alcohol [61], it can also be used alone through several chemical modifications. However, the swelling ratio of the dextran hydrogels was not investigated, probably due to an increased difficulty in crosslinking [56,62]. Therefore, dextran is usually added as a secondary polymer within the continuous phase. In this context, the present study demonstrated the regenerative capacity of the hydrogels containing chitosan and dextran.

To further increase the antimicrobial and regenerative potential of the hydrogels, a dispersed phase consisting of either readily added or in situ formed magnetite nanoparticles was included. The hypothesis behind comparing the effects of readily added or in situ formed magnetite nanoparticles is based on two anticipated processes. On one hand, the in situ formation of magnetite nanoparticles is expected to be more uniform throughout the entire volume of the hydrogel, which would lead to an enhanced antimicrobial activity. On the other hand, as the reaction yield is generally less than the amount of the predicted product, it was expected that the magnetite formation process would not be complete. In this manner, the hydrogels would contain a mixture of in situ magnetite nanoparticles and iron ions distributed throughout the hydrogel, where they would form iron complexes with the polymeric chains. This is of interest because the mechanisms involved in the antimicrobial activity of nanoparticles involves the release of Fe^2+^ and Fe^3+^ ions within the microbial cell, which further leads to the production of reactive oxygen species and the consequent disruption of DNA replication, breakage of the DNA double-strand, and peroxidation of lipids [63,64,65,66,67]. As hypothesized, the antimicrobial results demonstrated a higher efficiency of the hydrogels containing the in situ magnetite due to the presence of the mixture of iron ions and magnetite nanoparticles. In this manner, a prolonged antimicrobial effect could be achieved through the initial release of the iron ions, followed by the prolonged release of the magnetite nanoparticles. Furthermore, the presence of glycerol does not significantly influence the antimicrobial activity of the hydrogels.

In regard to the cell viability results, HUVEC was chosen as the cell line in order to ensure the first step towards evaluating the skin regeneration potential. Thus, it was demonstrated that the addition of the dispersed phases promoted the cell proliferation processes, with no significant differences between the readily added and in situ formed magnetite nanoparticles. Moreover, glycerol further enhanced the proliferative potential, thus demonstrating the efficiency of the so obtained hydrogels as wound dressings for skin regeneration and concomitant prevention of infection.

## 5. Conclusions

The present study aimed to conduct a comparative study between the skin regeneration potential and the antimicrobial activities of four types of composite hydrogels. The continuous phase consisted of either chitosan-dextran or chitosan-dextran-glycerol, while the dispersed phase included either readily added and in situ formed magnetite nanoparticles. The results confirmed the porous structure of the hydrogels and a high swelling degree ranging between 300% (i.e., CS/D/G-5P hydrogel) and 850% (i.e., CS/D/G-1M hydrogel). Furthermore, the cell viability test demonstrated the proliferative potential of all samples, with increasingly higher values for the hydrogels containing the dispersed phase, followed by the hydrogels containing glycerol within the continuous phase. The antimicrobial activity demonstrated significant microbial development reduction, especially for the hydrogels containing the in situ formed magnetite. Among the synthesized hydrogels, the ones containing glycerol enhanced the cell viability, but slightly decreased the antimicrobial activity; thus, the hydrogel with the optimal proliferative and antimicrobial properties would be CS/D/G-10P. In this manner, the present study successfully developed a series of hydrogels that could effectively be used as wound dressings for skin regeneration and microbial contamination prevention. 

## Figures and Tables

**Table 1:** Summary of the composite hydrogels obtained and their composition.

Hydrogel	Magnetite Nanoparticles (%)	Magnetite Precursors (%)	Dextran Solvent
CS/D	-	-	water
CS/D-1P	-	1	
CS/D-5P	-	5	
CS/D-10P	-	10	
CS/D-1M	1	-	
CS/D-5M	5	-	
CS/D-10M	10	-	
CS/D/G	-	-	glycerol
CS/D/G-1P	-	1	
CS/D/G-5P	-	5	
CS/D/G-10P	-	10	
CS/D/G-1M	1	-	
CS/D/G-5M	5	-	
CS/D/G-10M	10	-	

**Table 2:** Bonds assigned to the absorption bands registered within the FT-IR spectra.

Bond Type	Wavenumber (cm^−1^)
O-H stretching	3600–3100
C-H symmetric	2917
C-H asymmetric	2877
CH_2_ bending	1417
CH_3_ symmetrical deformation	1370
asymmetric C-O-C stretching	1149
C-O stretching	1012
C=O stretching	1651
N-H bending	1567
Fe-O	541

**Table 3:** Antimicrobial activity of hydrogel samples expressed as logarithmic reduction values.

	CS/D	CS/D-1P	CS/D-5P	CS/D-10P	CS/D-1M	CS/D-5M	CS/D-10M	CS/D/G	CS/D/G-1P	CS/D/G-5P	CS/D/G-10P	CS/D/G-1M	CS/D/G-5M	CS/D/G-10M
*S. aureus*	1.48 ± 0.11	1.42 ± 0.06	1.54 ± 0.21	1.64 ± 0.26	1.43 ± 0.14	1.42 ± 0.06	1.16 ± 0.17	1.15 ± 0.09	1.29 ± 0.08	0.99 ± 0.07	1.40 ± 0.28	1.24 ± 0.24	1.24 ± 0.18	1.34 ± 0.15
*Ps. aeruginosa*	1.48 ± 0.05	1.58 ± 0.20	1.42 ± 0.03	1.42 ± 0.03	1.44 ± 0.10	1.60 ± 0.03	1.42 ± 0.03	1.38 ± 0.06	1.48 ± 0.05	1.38 ± 0.06	1.30 ± 0.07	1.38 ± 0.06	1.62 ± 0.31	1.44 ± 0.01
*C. albicans*	0.58 ± 0.22	0.72 ± 0.12	0.82 ± 0.02	0.81 ± 0.03	0.58 ± 0.22	0.72 ± 0.12	0.74 ± 0.12	0.78 ± 0.06	0.79 ± 0.06	0.57 ± 0.03	0.81 ± 0.03	0.72 ± 0.12	0.82 ± 0.03	0.80 ± 0.03

**Table 4:** Antimicrobial activity of hydrogel samples expressed as percentual reduction values.

	CS/D	CS/D-1P	CS/D-5P	CS/D-10P	CS/D-1M	CS/D-5M	CS/D-10M	CS/D/G	CS/D/G-1P	CS/D/G-5P	CS/D/G-10P	CS/D/G-1M	CS/D/G-5M	CS/D/G-10M
*S. aureus*	96.5 ± 0.95	96.1 ± 0.49	96.7 ± 1.55	97.2 ± 1.80	96.1 ± 1.38	96.1 ± 0.49	92.5 ± 2.46	92.82 ± 1.54	94.72 ± 1.00	89.72 ± 1.62	95.31 ± 2.22	93.49 ± 2.88	93.84 ± 2.11	95.21 ± 1.53
*Ps. aeruginosa*	96.6 ± 0.40	97.1 ± 1.05	96.1 ± 0.27	96.1 ± 0.27	96.2 ± 0.89	97.4 ± 0.18	96.1 ± 0.27	95.78 ± 0.56	96.66 ± 0.40	95.78 ± 0.56	94.98 ± 0.83	95.78 ± 0.56	96.95 ± 1.87	96.38 ± 0.07
*C. albicans*	70.2 ± 14.96	80.36 ± 5.36	84.67 ± 0.80	84.4 ± 0.96	70.2 ± 16.96	80.3 ± 5.36	81.1 ± 5.33	83.03 ± 2.56	83.42 ± 2.44	73.21 ± 1.79	84.53 ± 1.13	80.36 ± 5.36	84.66 ± 0.95	84.16 ± 1.26

## Supplementary Materials

The following supporting information can be downloaded at: https://www.mdpi.com/article/10.3390/pharmaceutics14122620/s1, Figure S1: Schematic diagram of the methodology employed for the synthesis of the composite hydrogels; Figure S2: Pictures of the hydrogels after the lyophilization process; Figure S3: Elemental distribution within the CS/D hydrogels; Figure S4: Elemental distribution within the CS/D/G hydrogels.

## References

[1] Hombach-Klonisch S., Klonisch T., Peeler J. (2019). Sobotta Clinical Atlas of Human Anatomy.

[2] Neacsu I., Leau S., Marin S., Holban A., Vasile B., Nicoară A., Ene V., Bleotu C., Kaya M., Ficai A. (2021). Collagen-carboxymethylcellulose biocomposite wound-dressings with antimicrobial activity. Materials.

[3] Di Meglio P., Conrad C., Ratcliffe M.J.H. (2016). Psoriasis, cutaneous lupus erithematosus and immunobiology of the skin. Encyclopedia of Immunobiology.

[4] Yamate J., Suttie A.W. (2018). The skin and subcutis. Boorman’s Pathology of the Rat.

[5] Jeschke M.G., van Baar M.E., Choudhry M.A., Chung K.K., Gibran N.S., Logsetty S. (2020). Burn injury. Nat. Rev. Dis. Prim..

[6] Sen C.K. (2021). Human wound and its burden: Updated 2020 compendium of estimates. Adv. Wound Care.

[7] Järbrink K., Ni G., Sönnergren H., Schmidtchen A., Pang C., Bajpai R., Car J. (2017). The humanistic and economic burden of chronic wounds: A protocol for a systematic review. Syst. Rev..

[8] Guo Y., Bae J., Fang Z., Li P., Zhao F., Yu G. (2020). Hydrogels and hydrogel-derived materials for energy and water sustainability. Chem. Rev..

[9] Wang W., Narain R., Zeng H., Narain R. (2020). Hydrogels. Polymer Science and Nanotechnology.

[10] Peppas N.A., Hoffman A.S., Wagner W.R., Sakiyama-Elbert S.E., Zhang G., Yaszemski M.J. (2020). 1.3.2e—Hydrogels. Biomaterials Science.

[11] Choudhary B., Paul S.R., Nayak S.K., Qureshi D., Pal K., Pal K., Banerjee I. (2018). 11—Synthesis and biomedical applications of filled hydrogels. Polymeric Gels.

[12] Korah L.V., Anilkumar G., Thomas S., Thomas S., Balakrishnan P., Sreekala M.S. (2018). 5—Hydrogels, DNA, and rna polypeptides for the preparation of biomaterials. Fundamental Biomaterials: Polymers.

[13] Fan F., Saha S., Hanjaya-Putra D. (2021). Biomimetic hydrogels to promote wound healing. Front. Bioeng. Biotechnol..

[14] Sánchez-Cid P., Jiménez-Rosado M., Romero A., Pérez-Puyana V. (2022). Novel trends in hydrogel development for biomedical applications: A review. Polymers.

[15] Reddy M.S.B., Ponnamma D., Choudhary R., Sadasivuni K.K. (2021). A comparative review of natural and synthetic biopolymer composite scaffolds. Polymers.

[16] Eivazzadeh-Keihan R., Noruzi E.B., Mehrban S.F., Aliabadi H.A.M., Karimi M., Mohammadi A., Maleki A., Mahdavi M., Larijani B., Shalan A.E. (2022). Review: The latest advances in biomedical applications of chitosan hydrogel as a powerful natural structure with eye-catching biological properties. J. Mater. Sci..

[17] Aranaz I., Alcántara A.R., Civera M.C., Arias C., Elorza B., Heras Caballero A., Acosta N. (2021). Chitosan: An overview of its properties and applications. Polymers.

[18] Kou S., Peters L., Mucalo M. (2022). Chitosan: A review of molecular structure, bioactivities and interactions with the human body and micro-organisms. Carbohydr. Polym..

[19] Reshad R.A.I., Jishan T.A., Chowdhury N.N. (2021). Chitosan and its broad applications: A brief review. J. Clin. Exp. Investig..

[20] Zarrintaj P., Saeb M.R., Jafari S.H., Mozafari M., Ajitha A.R., Thomas S. (2020). Application of compatibilized polymer blends in biomedical fields. Compatibilization of Polymer Blends.

[21] Annu, Ahmed S., Ahmed S. (2021). 1—Advanced green materials: An overview. Advanced Green Materials.

[22] Varghese S.A., Rangappa S.M., Siengchin S., Parameswaranpillai J., Chen Y. (2020). Natural polymers and the hydrogels prepared from them. Hydrogels Based on Natural Polymers.

[23] Goyal S., Hernández N.B., Cochran E.W. (2021). An update on the future prospects of glycerol polymers. Polym. Int..

[24] (2019). M100 Performance Standards for Antimicrobial Susceptibility Testing a CLSI Supplement for Global Application. https://clsi.org/standards/products/microbiology/documents/m100/.

[25] Mostafavi M., Tucker A., Hsieh S. (2017). Intramolecular hydrogen-bonding effects on o-h stretch overtone excitation for fluorinated hydroperoxides. Chem. Phys..

[26] Glišić S., Nikolić G., Cakic M., Trutic N. (2015). Spectroscopic study of copper(ii) complexes with carboxymethyl dextran and dextran sulfate. Russ. J. Phys. Chem. A.

[27] Queiroz M., Melo K., Sabry D., Sassaki G., Rocha H. (2014). Does the use of chitosan contribute to oxalate kidney stone formation?. Mar. Drugs.

[28] Krishnaveni B., Ragunathan R. (2015). Extraction and characterization of chitin and chitosan from f.Solani cbnr bkrr, synthesis of their bionanocomposites and study of their productive application. J. Pharm. Sci. Res..

[29] Chircov C., Ștefan R.-E., Dolete G., Andrei A., Holban A.M., Oprea O.-C., Vasile B.S., Neacșu I.A., Tihăuan B. (2022). Dextran-coated iron oxide nanoparticles loaded with curcumin for antimicrobial therapies. Pharmaceutics.

[30] Chircov C., Bîrcă A.C., Vasile B.S., Oprea O.-C., Huang K.-S., Grumezescu A.M. (2022). Microfluidic synthesis of -nh2- and -cooh-functionalized magnetite nanoparticles. Nanomaterials.

[31] Chircov C., Bîrcă A.C., Grumezescu A.M., Vasile B.S., Oprea O., Nicoară A.I., Yang C.-H., Huang K.-S., Andronescu E. (2021). Synthesis of magnetite nanoparticles through a lab-on-chip device. Materials.

[32] Chircov C., Matei M.-F., Neacșu I.A., Vasile B.S., Oprea O.-C., Croitoru A.-M., Trușcă R.-D., Andronescu E., Sorescu I., Bărbuceanu F. (2021). Iron oxide–silica core–shell nanoparticles functionalized with essential oils for antimicrobial therapies. Antibiotics.

[33] Ruffin M., Brochiero E. (2019). Repair process impairment by pseudomonas aeruginosa in epithelial tissues: Major features and potential therapeutic avenues. Front. Cell Infect. Microbiol..

[34] Aziz M.A., Cabral J.D., Brooks H.J., Moratti S.C., Hanton L.R. (2012). Antimicrobial properties of a chitosan dextran-based hydrogel for surgical use. Antimicrob. Agents Chemother..

[35] Liao N., Unnithan A.R., Joshi M.K., Tiwari A.P., Hong S.T., Park C.-H., Kim C.S. (2015). Electrospun bioactive poly (ε-caprolactone)–cellulose acetate–dextran antibacterial composite mats for wound dressing applications. Colloids Surf. A Physicochem. Eng. Asp..

[36] Flynn J., Culebras M., Collins M.N., Hudson S.P. (2022). The impact of varying dextran oxidation levels on the inhibitory activity of a bacteriocin loaded injectable hydrogel. Drug Deliv. Transl. Res..

[37] Qiu H., Si Z., Luo Y., Feng P., Wu X., Hou W., Zhu Y., Chan-Park M.B., Xu L., Huang D. (2020). The mechanisms and the applications of antibacterial polymers in surface modification on medical devices. Front. Bioeng. Biotechnol..

[38] Alqahtani F., Aleanizy F., El Tahir E., Alhabib H., Alsaif R., Shazly G., AlQahtani H., Alsarra I., Mahdavi J. (2020). Antibacterial activity of chitosan nanoparticles against pathogenic n. Gonorrhoea. Int. J. Nanomed..

[39] Madureira A.R., Pereira A., Pintado M. (2015). Current state on the development of nanoparticles for use against bacterial gastrointestinal pathogens. Focus on chitosan nanoparticles loaded with phenolic compounds. Carbohydr. Polym..

[40] Basseri H., Bakhtiyari R., Hashemi S.J., Baniardelani M., Shahraki H., Hosainpour L. (2019). Antibacterial/antifungal activity of extracted chitosan from american cockroach (dictyoptera: Blattidae) and german cockroach (blattodea: Blattellidae). J. Med. Entomol..

[41] Lagat M.K., Were S., Ndwigah F., Kemboi V.J., Kipkoech C., Tanga C.M. (2021). Antimicrobial activity of chemically and biologically treated chitosan prepared from black soldier fly (hermetia illucens) pupal shell waste. Microorganisms.

[42] Shin C.-S., Kim D.-Y., Shin W.-S. (2019). Characterization of chitosan extracted from mealworm beetle (tenebrio molitor, zophobas morio) and rhinoceros beetle (allomyrina dichotoma) and their antibacterial activities. Int. J. Biol. Macromol..

[43] Su X., Zivanovic S., D’souza D.H. (2009). Effect of chitosan on the infectivity of murine norovirus, feline calicivirus, and bacteriophage ms2. J. Food Prot..

[44] Milewska A., Chi Y., Szczepanski A., Barreto-Duran E., Dabrowska A., Botwina P., Obloza M., Liu K., Liu D., Guo X. (2021). Htcc as a polymeric inhibitor of sars-cov-2 and mers-cov. J. Virol..

[45] Lo W.-H., Deng F.-S., Chang C.-J., Lin C.-H. (2020). Synergistic antifungal activity of chitosan with fluconazole against candida albicans, candida tropicalis, and fluconazole-resistant strains. Molecules.

[46] Chabra A., Rahimi-Esboei B., Habibi E., Monadi T., Azadbakht M., Elmi T., valian H.K., Akhtari J., Fakhar M., Naghshvar F. (2019). Effects of some natural products from fungal and herbal sources on giardia lamblia in vivo. Parasitology.

[47] Abdel-Latif M., El-Shahawi G., Aboelhadid S., Abdel-Tawab H. (2017). Immunoprotective effect of chitosan particles on hymenolepis nana–infected mice. Scand. J. Immunol..

[48] Yan D., Li Y., Liu Y., Li N., Zhang X., Yan C. (2021). Antimicrobial properties of chitosan and chitosan derivatives in the treatment of enteric infections. Molecules.

[49] Matica M.A., Aachmann F.L., Tøndervik A., Sletta H., Ostafe V. (2019). Chitosan as a wound dressing starting material: Antimicrobial properties and mode of action. Int. J. Mol. Sci..

[50] Sahariah P., Másson M. (2017). Antimicrobial chitosan and chitosan derivatives: A review of the structure-activity relationship. Biomacromolecules.

[51] Feng P., Luo Y., Ke C., Qiu H., Wang W., Zhu Y., Hou R., Xu L., Wu S. (2021). Chitosan-based functional materials for skin wound repair: Mechanisms and applications. Front. Bioeng. Biotechnol..

[52] Olicón-Hernández D.R., Hernández-Lauzardo A.N., Pardo J.P., Peña A., Velázquez-del Valle M.G., Guerra-Sánchez G. (2015). Influence of chitosan and its derivatives on cell development and physiology of ustilago maydis. Int. J. Biol. Macromol..

[53] Garcia L.G.S., Guedes G.M.M., da Silva M.L.Q., Castelo-Branco D., Sidrim J.J.C., Cordeiro R.A., Rocha M.F.G., Vieira R.S., Brilhante R.S.N. (2018). Effect of the molecular weight of chitosan on its antifungal activity against candida spp. In planktonic cells and biofilm. Carbohydr. Polym..

[54] Mamidi N., Delgadillo R.M.V. (2021). Design, fabrication and drug release potential of dual stimuli-responsive composite hydrogel nanoparticle interfaces. Colloids Surf. B Biointerfaces.

[55] Zhu Q., Jiang M., Liu Q., Yan S., Feng L., Lan Y., Shan G., Xue W., Guo R. (2018). Enhanced healing activity of burn wound infection by a dextran-ha hydrogel enriched with sanguinarine. Biomater. Sci..

[56] Sun G., Zhang X., Shen Y.I., Sebastian R., Dickinson L.E., Fox-Talbot K., Reinblatt M., Steenbergen C., Harmon J.W., Gerecht S. (2011). Dextran hydrogel scaffolds enhance angiogenic responses and promote complete skin regeneration during burn wound healing. Proc. Natl. Acad. Sci. USA.

[57] Zhou L., Zhou L., Wei C., Guo R. (2022). A bioactive dextran-based hydrogel promote the healing of infected wounds via antibacterial and immunomodulatory. Carbohydr. Polym..

[58] Lin S.-P., Kung H.-N., Tsai Y.-S., Tseng T.-N., Hsu K.-D., Cheng K.-C. (2017). Novel dextran modified bacterial cellulose hydrogel accelerating cutaneous wound healing. Cellulose.

[59] Garg A., Garg S., Shukla A., Janadri S., Kori M., Lodhi S. (2022). Fabrication and evaluation of carboxy methyl cellulose anchored dextran bioinspired hydrogel for effective delivery of piroxicam. Indian J. Pharm. Sci..

[60] Mansuroğlu B., Kızılbey K., Şayan Poyraz F., Yurttaş Z., Fuerkaiti S.N., Abaoğlu I.Y., Başat H.N. (2021). Chitosan/dextran sulphate sodium hydrogels for wound healing material: Preparation, characterisation and in vitro evaluation. Mater. Technol..

[61] Lin S.-P., Lo K.-Y., Tseng T.-N., Liu J.-M., Shih T.-Y., Cheng K.-C. (2019). Evaluation of pva/dextran/chitosan hydrogel for wound dressing. Cell. Polym..

[62] Qiu X., Zhang J., Cao L., Jiao Q., Zhou J., Yang L., Zhang H., Wei Y. (2021). Antifouling antioxidant zwitterionic dextran hydrogels as wound dressing materials with excellent healing activities. ACS Appl. Mater. Interfaces.

[63] Spirescu V.A., Chircov C., Grumezescu A.M., Vasile B.Ș., Andronescu E. (2021). Inorganic nanoparticles and composite films for antimicrobial therapies. Int. J. Mol. Sci..

[64] Chaudhary R.G., Bhusari G.S., Tiple A.D., Rai A.R., Somkuvar S.R., Potbhare A.K., Lambat T.L., Ingle P.P., Abdala A.A. (2019). Metal/metal oxide nanoparticles: Toxicity, applications, and future prospects. Curr. Pharm. Des..

[65] Ye Q., Chen W., Huang H., Tang Y., Wang W., Meng F., Wang H., Zheng Y. (2020). Iron and zinc ions, potent weapons against multidrug-resistant bacteria. Appl. Microbiol. Biotechnol..

[66] Abbas H.S., Krishnan A. (2020). Magnetic nanosystems as a therapeutic tool to combat pathogenic fungi. Adv. Pharm. Bull..

[67] Gabrielyan L., Hovhannisyan A., Gevorgyan V., Ananyan M., Trchounian A. (2019). Antibacterial effects of iron oxide (Fe_3_O_4_) nanoparticles: Distinguishing concentration-dependent effects with different bacterial cells growth and membrane-associated mechanisms. Appl. Microbiol. Biotechnol..

