# Supplementary material for: Chitosan-Dextran-Glycerol Hydrogels Loaded with Iron Oxide Nanoparticles for Wound Dressing Applications
Source: Pharmaceutics. 2022 Nov 28;14(12):2620. doi: 10.3390/pharmaceutics14122620 (PMC9784071; doi:10.3390/pharmaceutics14122620)
Supplement: Supplementary file 1 [file pharmaceutics-14-02620-s001.zip › pharmaceutics-2043283-supplementary.pdf]

Supplementary Materials

Chitosan-Dextran-Glycerol Hydrogels Loaded with Iron Oxide Nanoparticles for Wound Dressing Applications

Figure S1 describes the methodology employed for the synthesis of the hydrogels, while Figure S2 shows the pictures of the hydrogels taken after the lyophilization process.

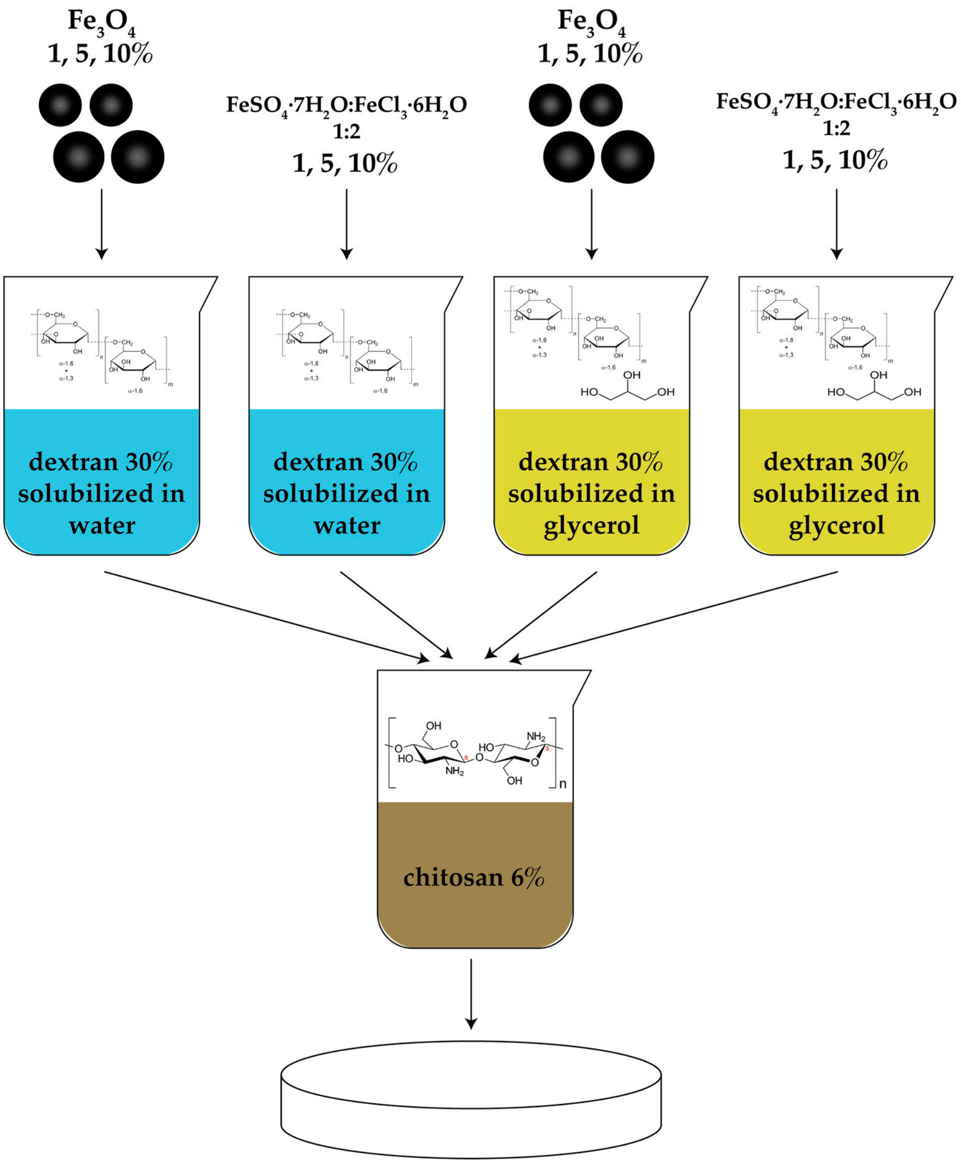

Figure S1: Schematic diagram of the methodology employed for the synthesis of the composite hydrogels.

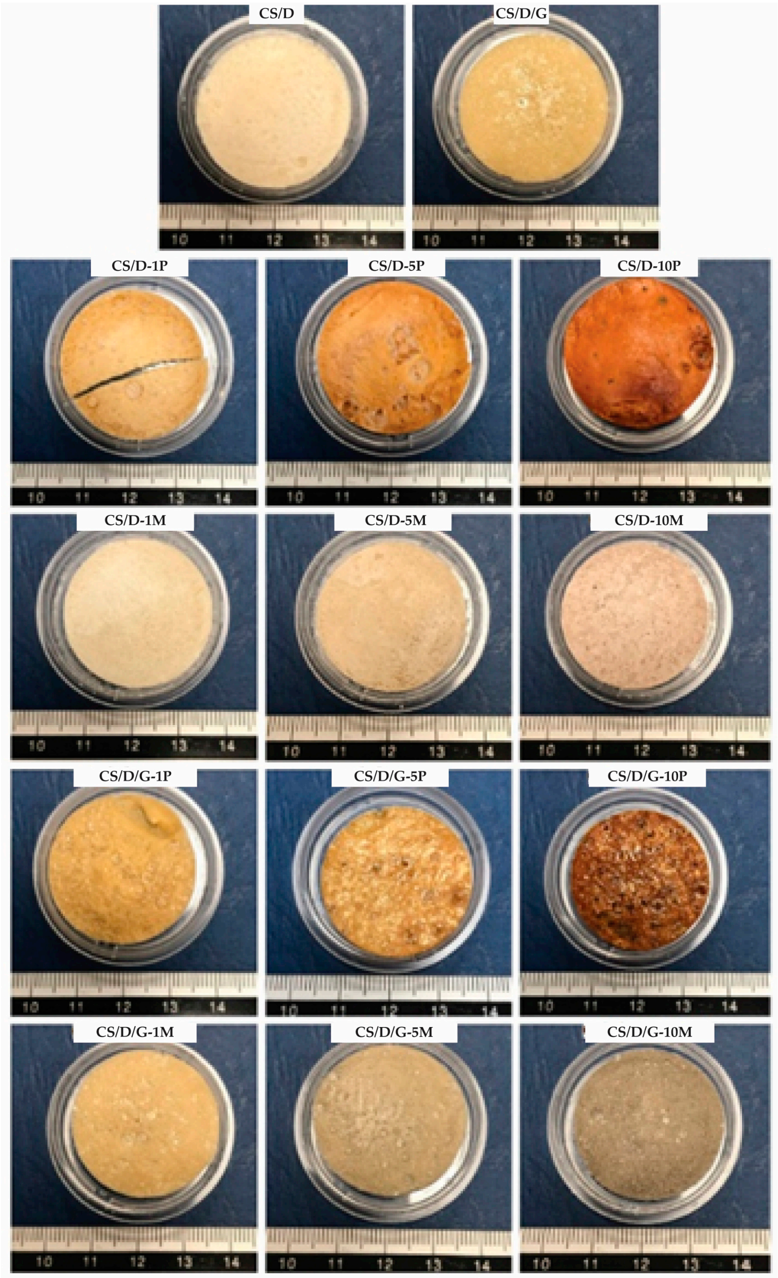

Figure S2: Pictures of the hydrogels after the lyophilization process.

Figure S3 and S4 present the elemental distribution within the hydrogels.

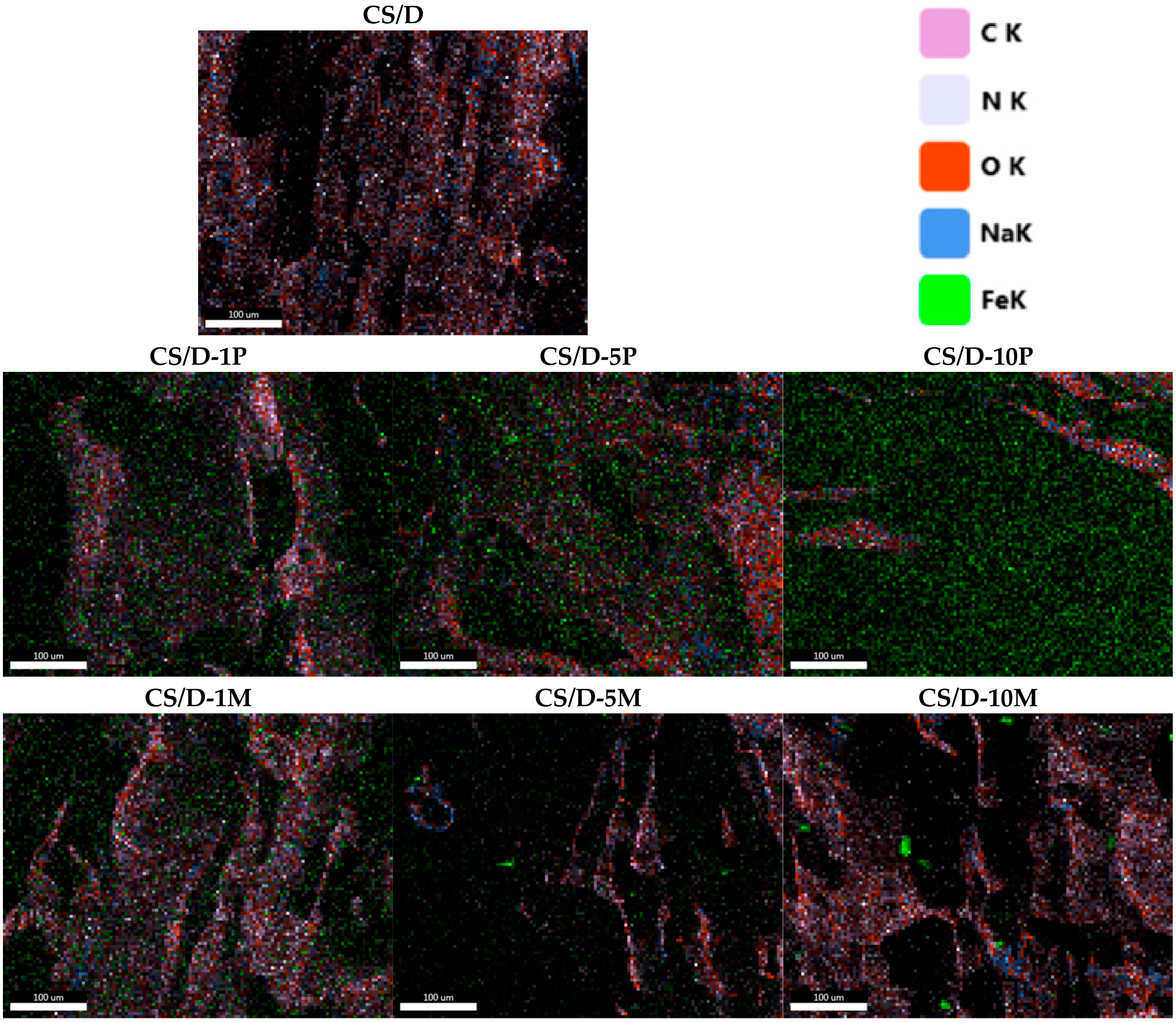

Figure S3: Elemental distribution within the CS/D hydrogels.

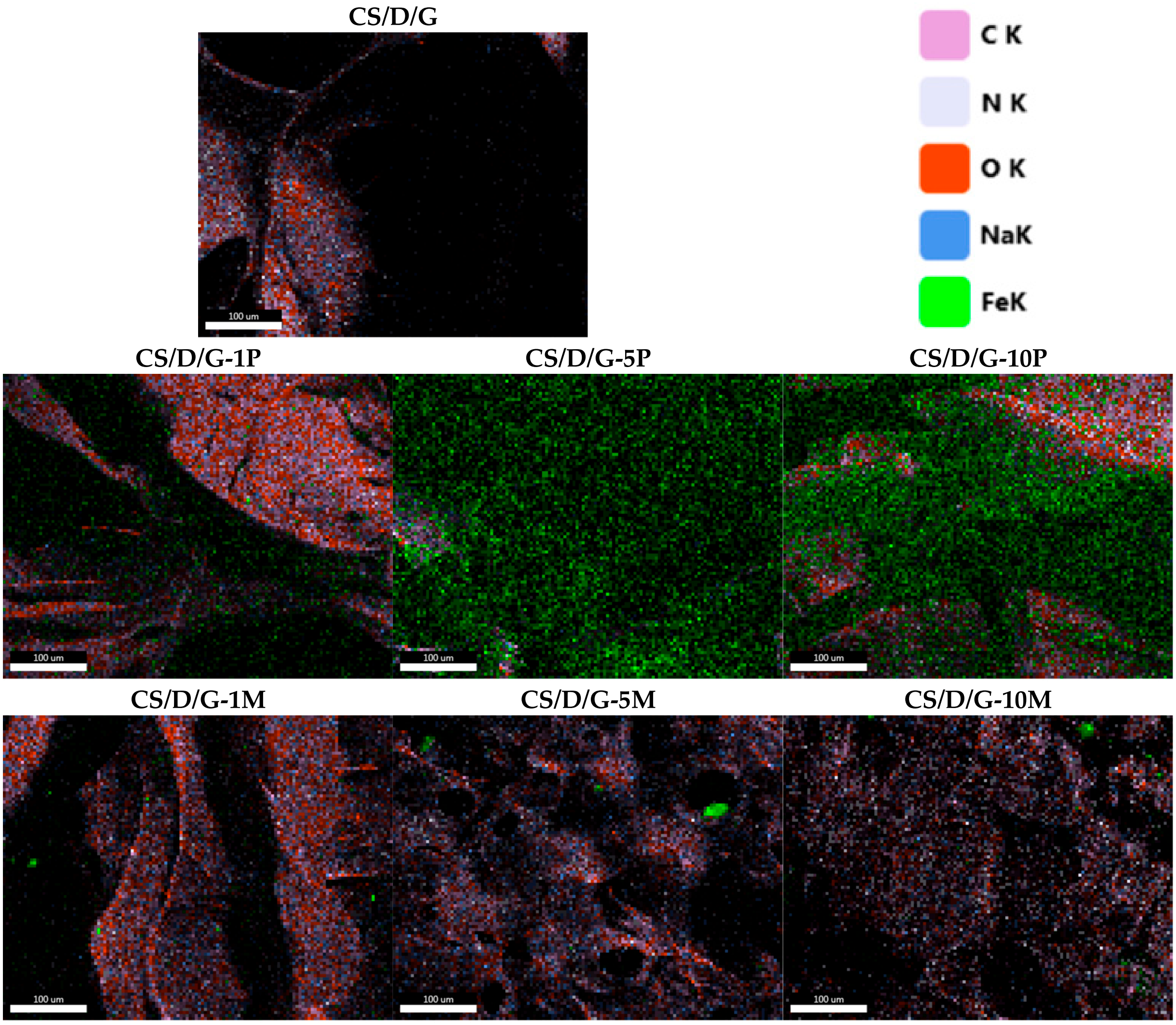

Figure S4: Elemental distribution within the CS/D/G hydrogels.
